# Undertreatment and Underachievement of LDL-C Target among Individuals with High and Very High Cardiovascular Risk in the Malaysian Community

**DOI:** 10.3390/healthcare10122448

**Published:** 2022-12-05

**Authors:** Aimi Zafira Razman, Noorhida Baharudin, Noor Alicezah Mohd Kasim, Alyaa Al-Khateeb, Zaliha Ismail, Hapizah Nawawi

**Affiliations:** 1 Institute of Pathology, Laboratory and Forensic Medicine (I-PPerForM), Universiti Teknologi MARA, Sungai Buloh 47000, Selangor, Malaysia; 2 Department of Primary Care Medicine, Faculty of Medicine, Universiti Teknologi MARA, Sungai Buloh 47000, Selangor, Malaysia; 3 Department of Pathology, Faculty of Medicine, Universiti Teknologi MARA, Sungai Buloh 47000, Selangor, Malaysia; 4 UiTM Al-Sultan Abdullah Hospital, Puncak Alam 42300, Selangor, Malaysia; 5 Department of Biochemistry and Molecular Medicine, Faculty of Medicine, Universiti Teknologi MARA, Sungai Buloh 47000, Selangor, Malaysia; 6 Department of Public Health Medicine, Faculty of Medicine, Universiti Teknologi MARA, Sungai Buloh 47000, Selangor, Malaysia

**Keywords:** dyslipidaemia, very high risk, high risk, lipid lowering therapy, low density

## Abstract

Dyslipidaemia is a major cause of morbidity and mortality. The aims of this study are to determine the prevalence of dyslipidaemia subtypes, the proportions of lipid-lowering therapy (LLT) use, and the achievement of low-density lipoprotein cholesterol (LDL-C) treatment targets for high-risk (HR) and very high-risk (VHR) Malaysians. This cross-sectional study involves 5279 participants across 11 states in Malaysia. The data were obtained through a standardised questionnaire, anthropometric measurements, venous glucose and lipid profile. The participants with existing cardiovascular disease (CVD) or diabetes with at least one of the other major risk factors (smoking, hypertension or dyslipidaemia) were grouped into the VHR category. Other participants were risk-categorised using the Framingham General CVD Risk Score (FRS-CVD). The prevalence of elevated LDL-C, LLT use and LDL-C target were set according to respective risk categories. Pearson’s chi-squared test was used to test the difference in the proportions. The mean ± standard deviation (SD) age was 41.1 ± 14.8 years, and 62.2% (3283/5279) of the group were females. Within the participant group, 51.5% were found to have elevated total cholesterol, 28.8% had low HDL-C, and 33.8% had high triglyceride. As for elevated LDL-C, 9.8% were in VHR, 8.6% in HR, 5.8% in MR and 34.9% in LR categories. Among the VHR group, 75.8% were not on LLT, and only 15.9% achieved the LDL-C target. As for the HR category, 87.7% were not on LLT, and only 16.1% achieved the LDL-C target. Dyslipidaemia is highly prevalent among Malaysians. The majority of VHR and HR participants were not on LLT and did not achieve LDL-C treatment targets. Proactive programs are warranted to combat dyslipidaemia-associated CVD events in these groups.

## 1. Introduction

Cardiovascular disease (CVD) is one of the leading causes of death globally [1]. According to the World Health Organisation (WHO), about 18 million individuals die annually from CVD, particularly coronary artery disease (CAD) and stroke, which account for 32% of all global death [2]. Dyslipidaemia is a major modifiable risk factor for CVD. It can be defined as one or any combination of high total cholesterol (TC), high low-density lipoprotein cholesterol (LDL-C), high triglycerides (TG) and low high-density lipoprotein cholesterol (HDL-C) levels [2]. Epidemiological studies have established that elevated TC and LDL-C are strongly related to an increased CAD risk in many populations worldwide [3,4,5,6]. Tall et al. (2022) discovered that the elevation of TG level is independently associated with CAD, together with a low HDL-C level [7]. Furthermore, elevated TG and low HDL-C are associated with atherogenic dyslipidaemia [8].

The World Health Organization (2008) reported that the prevalence of hypercholesterolaemia (HC) was highest in the European region (54%), followed by the American (48%), while the lowest prevalence was seen in the African (22.6%) and the Southeast Asian regions (29.0%) [9,10]. Lin et al. (2018) reported that the prevalence of dyslipidaemia subtypes varied across Asia Pacific regions, with the prevalence of TC ranging from 9% to 47%, elevated LDL-C ranging from 7% to 48%, elevated TG ranging from 9% to 39%, and low HDL-C ranging from 5% to 72% [11].

In Malaysia, the National Health and Morbidity Survey (NHMS) reported an increasing trend of HC from the years of 2011 (35.1%) to 2015 (47.7%) [12]. In another study by Mohamed-Yassin et al. (2021), the prevalence of dyslipidaemia among adults was high, of which 64% and 56.7% of Malaysians had elevated TC and LDL-C, respectively [13]. The prevalence of HC and low HDL-C among the Malays in rural areas in Malaysia were also surprisingly high (67.3% and 13.1%, respectively), which may be attributed to the unhealthy lifestyle trend observed among these communities [14]. 

Various local and international guidelines recommend LDL-C as the primary target for lipid-lowering therapy (LLT) [2,15,16]. Reduction of LDL-C with a statin, in addition to therapeutic lifestyle intervention, remains the cornerstone in dyslipidaemia management to reduce the CVD risk [2,17], in which 1 mmol/L of reduction in LDL-C reduces the CVD mortality by 22% [18]. In addition, recent international guidelines recommended a healthy diet as the cornerstone of CVD prevention in all individuals [19]. These guidelines also emphasised the stratification of an individual’s CVD risk, which would guide the intensity of LLT and determine the LDL-C treatment targets [2,17,18,19,20]. The Framingham General CVD (FRS-CVD) risk score is a risk stratification tool which has been widely used and validated among the multiethnic population in Malaysia [21]. According to the Malaysian Clinical Practice Guideline (CPG) on the Management of Dyslipidaemia, very high-risk (VHR) individuals include those with an existing CVD or diabetes with at least one of the other major risk factors (smoking, hypertension or dyslipidaemia). Those without existing CVD were risk-stratified using the FRS-CVD tool. The high-risk (HR) individuals include those with >20% of a 10-year CVD risk by the FRS-CVD score [17]. Several previous studies have examined the use of LLT and targeted LDL-C reduction in HR groups [21,22,23,24]. However, despite LLT, the majority of HR individuals did not achieve their LDL-C goals [25,26,27,28].

Dyslipidaemia has been established as a major risk factor for CVD; therefore, the data on prevalence, treatment and therapeutic target achievements among individuals in the community is vital for monitoring and developing new strategies for CVD prevention, nationally and globally. The prevalence of dyslipidaemia in Malaysia has been previously described by other local studies [13,14]. However, to the best of our knowledge, there has been no report on the prevalence of elevated LDL-C according to CVD risk categories. While the prevalence of LLT among Malaysian and HR individuals was previously described by local studies [29,30], the literature regarding the achievements of LDL-C treatment targets according to risk categories remains scarce. Thus, this study aimed to determine the prevalence of dyslipidaemia subtypes, including elevated LDL-C according to risk categories, the proportions of LLT use and the achievement of LDL-C treatment targets for HR and VHR individuals.

## 2. Materials and Methods

### 2.1. Study Design and Population

This investigation was a cross-sectional study from Malaysian Health and Wellbeing Assessment for Coronary Risk Epidemiological Study (MyHEBAT-CRES). The method was described in previous literature [31]. The participants were recruited through nationwide health screening programs conducted in various regions of the country, including East and West Malaysia, from 2011 to 2018. The ethical approval was obtained from the Research Ethics Committee of Universiti Teknologi MARA (Reference number: UiTM 600-RMl [5/1/6)]) prior to the commencement of the study, following the Declaration of Helsinki.

All participants were recruited through health screening programs in 11 out of 14 states in East and West Malaysia, which were; Selangor, Kuala Lumpur, Pahang, Terengganu, Kelantan, Melaka, Kedah, Perak, Johor, Sabah and Sarawak. The health screening programs were held in school halls or general-purpose public halls during the weekends. The events were advertised by the local authority through social media and banners at least two weeks prior to the event. During registration, all participants were given a study information sheet and were screened for eligibility based on the inclusion and exclusion criteria. The inclusion criteria were: Malaysian and aged between 18 and 75 years old, while participants with secondary HC (hypothyroidism, chronic kidney disease, nephrotic syndrome and cholelithiasis) or pregnancy were excluded from the study. Those who were eligible were provided with written informed consent prior to the enrolment into the study.

### 2.2. Study Procedures

All interviewers and investigators were trained on the study procedure before the commencement of the study. Interviews via standardised questionnaires were used to gather participants’ demographic information and personal clinical history (presence of comorbidities, smoking status, LLT use and family history (premature coronary artery disease [PCAD])). Physical examination findings, including anthropometric measurements such as weight, height, body mass index (BMI), waist circumference (WC) and hip circumference (HC), were recorded on-site. Blood pressure was measured twice, two minutes apart, in a sitting position using automatic digital blood pressure (BP) monitors (Omron HEM-8712, Japan). The average of the last two systolic and diastolic measurements was used as the BP reading for the individual participants.

### 2.3. Blood Sampling and Laboratory Analysis

A total of 3 mL venous blood samples were collected in the plain and fluoride oxalate tubes. All biochemical analyses for serum TC, TG, HDL-C and plasma glucose were analysed using an automated biochemical analyser (COBAS Integra^®^ 400, Roche Holding AG; Basel, Switzerland). The LDL-C concentration was calculated using the Friedewald equation [32]. Calibration and internal quality control (QC) were performed according to the standardised pathology laboratory guideline, which complies with the ISO 15189 standard, certified by the National Laboratory Accreditation Scheme and recognised by Asia-Pacific Laboratory Accreditation Cooperation (APLAC) and International Laboratory Accreditation Cooperation (ILAC).

### 2.4. Definition of Terms

The word “urban” was defined as communities with a population >10,000, while “rural” was defined as <10,000 of the population [33]. Education attainment was categorised into four groups; (1) no formal education, (2) primary education, which was defined as schooling between ages 7 to 12 years old, (3) secondary education, which was defined as schooling between ages 13 to 17 years old and (4) tertiary education which was defined as any qualification attained from college or university. The BMI and central obesity were categorised according to the Malaysian CPG on Management of Obesity, 2004 and WHO for the Asian population [34,35,36]. Underweight was defined as BMI < 18.5 kg/m², normal as BMI 18.5–22.9 kg/m², overweight as BMI 23–27.4 kg/m² and obesity as BMI ≥ 27.5 kg/m². Central obesity was defined as a waist circumference (WC) of ≥90 cm for men and ≥80 cm for women. Diabetes was defined as having a fasting plasma glucose (FPG) ≥7.0 mmol/L or random plasma glucose (RPG) >11.1 mmol/L or self-reported diabetes or use of anti-diabetic medication [37]. Hypertension was defined as an elevation of systolic BP ≥ 140 mmHg or diastolic BP ≥ 90 mmHg based on the average of two readings during health screening or self-reported hypertension or use of anti-hypertensive medication [38]. Family history of premature CAD was defined as self-reported having a first-degree relative with CAD or sudden death before the age of 55 for men or 65 for women [39].

### 2.5. CV Risk Stratification, Dyslipidaemia Subtypes and LDL-C Target Levels

A VHR individual was defined as a participant with an existing CVD or diabetes with at least one of the other major risk factors (smoking, hypertension or dyslipidaemia). For those without existing CVD, their 10-year risk for CVD event was calculated using the FRS-CVD tool [40]. High-risk individuals included those with FRS scores that confer a 10-year risk for CVD >20%. The moderate risk (MR) individuals included those with FRS scores that confer a 10-year risk for CVD of 10–20%, while low-risk (LR) individuals included those with FRS scores that confer a 10-year CVD risk of <10% [17].

In this study, HC was defined as TC > 5.2 mmol/L; low HDL was defined as <1.0 mmol/L for males and <1.2 mmol/L for females and elevated TG was defined as >1.7 mmol/L [17]. Elevated LDL-C was defined according to the CVD risk profiles; LDL-C ≥ 1.8 mmol/L for VHR, LDL-C > 2.6 mmol/L for HR, LDL-C *≥* 3.0 mmol/L for MR and LR risk groups [17].

LLT was defined as self-reported use of any lipid-lowering medications. The attainment of the LDL-C target levels for each subject was assessed according to the 2017 Malaysian CPG Management of Dyslipidaemia guideline [17] after evaluating the individual’s CVD risks. For the LR and MR groups, the LDL-C target was <3.0 mmol/L, while for the HR and VHR groups, the LDL-C targets were ≤2.6 mmol/L and <1.8 mmol/L, respectively.

### 2.6. Statistical Analysis

All analyses were performed using the SPSS software version 27 (IBM, Armonk, NY, USA). Socio-demographic, clinical characteristics, anthropometric and biochemical data of the participants were described using descriptive statistics. The results were presented as percentages for categorical variables, while numerical variables were described with mean [±standard deviation (SD)]. Pearson’s chi-squared test was used to test the difference in the proportions, and a *p*-value of <0.05 was considered statistically significant.

## 3. Results

### 3.1. Participants Characteristics

A total of 5279 eligible adults aged ≥18 participated in this study. The socio-demographic characteristics of the participants are shown in Table 1. The participants’ mean age was 41.1 ± (14.8) years old; 62.2% were females, 72.6% were Malays, and 58.5% were from urban areas. Almost half of them received tertiary education (44.4%). The prevalence of diabetes was 9.5%, while 31.5% of the participants were hypertensive. About 2.7% of the participants had existing CVD, and 11.5% of the participants had a family history of PCAD. The mean ± SD levels of TC, TG, LDL-C and HDL-C were 5.2 ± 1.2 mmol/L, 1.4 ± 0.9 mmol/L, 3.2 ± 1.0 mmol/L and 1.3 ± 0.4 mmol/L, respectively. The proportions of HR and VHR were 9.5% and 11.3%, respectively.

### 3.2. The Overall Prevalence of Dyslipidaemia Subtypes

Table 2 shows the prevalence of dyslipidaemia subtypes among the participants. Overall, out of 5279, 51.5% were found to have HC, 28.8% had low HDL-C, and 33.8% had high TG. As for elevated LDL-C, 9.8% were in VHR (LDL-C ≥ 1.8 mmol/L), 8.6% in HR (LDL-C > 2.6 mmol/L), 5.8% in MR (LDL-C ≥ 3.0 mmol/L) and 34.9% in LR (LDL-C ≥ 3.0 mmol/L) categories. Among all participants, those between the age of 50 and 59 years old had higher proportions of HC (25.8%), elevated TG (26.4%) and elevated LDL-C (35.6% in the VHR group). Participants in the urban locality had a significantly higher prevalence of HC, elevated TG and elevated LDL-C compared to those in rural areas.

### 3.3. Proportions of LLT Use among HR and VHR Participants

Table 3 shows the proportions of LLT use and LDL-C target achievement according to the risk categories. Overall, the majority of participants in each risk category were not on LLT. Among the VHR group, almost 24% had previous CVD, and among those who had previous CVD, 30.8% were on LLT, and only 11% achieved LDL-C target levels. A significant proportion of participants in the HR group (87.7%) and VHR group (75.8%) were not on LLT. In addition, among those who were on LLT, the majority of them did not achieve LDL-C target levels, particularly among the HR (83.9%) and VHR (84.1%) groups.

## 4. Discussion

This MyHEBAT-CRES study aims to determine the prevalence of dyslipidaemia subtypes, including elevated LDL-C according to risk categories, the proportion of LLT use and the achievement of LDL-C treatment targets for HR and VHR individuals. To the best of our knowledge, this study is the first to report on the prevalence of elevated LDL-C and LDL-C target levels achievement among those taking LLT, according to the risk categories.

The prevalence of HC found in the present study was 51.5%, which is higher than the findings from the Malaysian National Health and Morbidity Survey (NHMS) in 2015 (47.7%) [12]. The Malaysian NHMS reported a rising trend of HC over the years, in which 32.6% had HC in 2011 [41]. The prevalence increased in the subsequent report in 2015, where 47.7% of Malaysians had HC [12]. The prevalence of HC reported by Mohamed-Yassin et al. (2022) was higher compared to the present study (64% vs. 51.5%) [13]. This lower prevalence may be explained by the minimum age used in the present study (≥18 years old). Compared to other Asian countries, the Malaysian community had a higher prevalence of HC compared to Indonesia (49.5%) [42] and China (47.8%) [43] but lower than India (63.8%) [44]. However, based on the WHO region report from the Global Burden of Disease 2016, the WHO South-East Asia region was reported to have a low prevalence of HC (30%), while the highest prevalence of HC was in the WHO European Region (54%) [45]. The prevalence of HC may vary in different populations subject to the socio-demographic area [46,47], and different cut-off values are used in various studies [40,41].

In terms of the prevalence of elevated TG, this study reported a prevalence of 33.8%. The prevalence of elevated TG found in this study was lower compared to the study by Mohamed-Yassin et al. (37.4%) [13] and Nawawi et al. (46.1%) [14]. Compared to other countries, the prevalence of elevated TG in this study was lower than in Thailand (33.8% vs. 38.6%) [47] and higher than in Australia (13.9%) [48] and Indonesia (24.9%) [49]. For low HDL-C, the prevalence reported in this study was 28.8% and was higher compared to other studies conducted by Nawawi et al. (13.1%) and Nazri et al. (9.2%) [14,50]. However, Mohamed-Yassin et al. (2021) reported a higher prevalence of low HDL-C (36.2%) [13]. The difference in the prevalence of low HDL-C could be attributed to the dietary intake and the physical activity of the participants, which may reflect the HDL-C levels [50,51].

The present study is the first to report on the prevalence of elevated LDL-C according to risk categories. Of the participants with elevated LDL-C, 9.8% came from the VHR (LDL-C ≥ 1.8 mmol/L), 8.6% from HR (LDL-C > 2.6 mmol/L), 5.8% from MR (LDL-C ≥ 3.0 mmol/L), and 34.9% from LR (LDL-C ≥ 3.0 mmol/L), groups. Mohamed-Yassin et al. (2021) reported the prevalence of elevated LDL-C using the cut-off LDL-C of >3.4 mmol/L (56.7%) [13]. Nawawi et al., who studied 609 Malays, also found a comparable prevalence of elevated LDL-C (57.2%) [14]. Among countries in the Asian region, some countries reported a lower prevalence of elevated LDL-C, which were Korea (19.2%), China (17.9%) and Singapore (15.2%) [52,53,54]. Thailand also reported a lower prevalence of elevated LDL-C (26.9%) [47]. However, the LDL-C cut-off, according to the study in Thailand, was set according to the risk categories outlined by the National Cholesterol Education Program Adult Treatment Panel (NCEP ATP) III [39]. The variation in the prevalence of elevated LDL-C reported in other studies could possibly be explained by various LDL-C cut-offs used by them.

Various approaches and tools were used for CVD risk assessment, such as FRS-CVD [40], the Systemic Coronary Risk Estimation (SCORE) [55], the American Heart Association (AHA)/American College of Cardiology (ACC) Pooled Cohort Equation [56] and the World Health Organization (WHO) [57]. A local study using ACC/AHA risk stratification found that the risk model overestimated CVD risk in the Malaysian population [21]. Thus, in this study, FRS-CVD was used for CV risk scoring and the estimation for a 10-year CV event, as it has the advantage of the population receiving no or little treatment at the beginning or during the study; better discriminator for multiethnic population, as well as simple and easy for routine use by healthcare providers [21]. The risk assessment is vital as the LDL-C treatment threshold, and target levels are determined based on the individual’s CVD risk category [2,15,16].

Lipid-lowering therapy is one of the strategies to reduce the CVD burden [58]. The local Malaysian guideline recommends LLT at the outset for all patients in the VHR group regardless of their LDL-C level [17]. Local Malaysian and international guidelines advocate that LDL-C target levels are achieved according to the risk categories [17,20]. Furthermore, a recent European guideline on CVD prevention proposed that the treatment goal for individuals can be personalised using a stepwise approach as the treatments intensify. Using this approach as a tool helps physicians and patients pursue these targets that fit patient profiles and preferences [19]. This study discovered that among the VHR participants, about 75.8% were not on LLT. Another local study by Baharudin et al. reported a comparable finding where 74.2% of those with existing CVD were also not using any LLT [30]. In addition, in the present study, the majority of participants on LLT did not achieve LDL-C target levels for their respective risk categories, at the time of evaluation. Another study conducted in Korea by Yang et al. (2020) demonstrated better results, where 56% of HR individuals were on LLT [59]. Among those, less than 50% achieved the LDL-C target levels according to the Korea National Guideline [60]. Bruckert et al. also reported poor LDL-C control among individuals in the HR and VHR groups, where only 19% achieved targets [61]. Although, the Malaysian healthcare system offers free public healthcare, including LLT, the use of LLT and the achievement of therapeutic LDL-C target levels were still suboptimal. The challenges and possible reasons contributing to suboptimal treatment may include statin side effects, low adherence and use of less potent statin for patients in the HR and VHR groups [62]. Baharudin et al. highlighted the need for the LLT to be prescribed appropriately based on the patient’s clinical condition. With the proper prescription of LLT, those medically eligible subjects with socio-demographic disadvantages will have equal access to LLT, particularly for common LLT, such as statins, which are free to all Malaysian attending government healthcare facilities [30]. Furthermore, the poor achievement of the therapeutic LDL-C target from this study, particularly among those in the HR and VHR categories, emphasised an urgent need to improve the awareness of the general public and healthcare providers on the importance of early detection and efficient treatment of dyslipidaemia.

The present study also discovered that the proportion of existing CVD was only 2.7%, and the proportion of individuals with diabetes was much lower than in another local large population study (9.5% vs. 17.5%) [12]. The low prevalence of CVD and diabetes in this study may be explained by the younger population studied (mean age of 41.1 years old), much younger than the national average of first onset of CVD at 58 years old [63]. In the present study, 34.5% of Malaysians were obese, and this proportion was slightly higher compared to the national data, 30.6% [12] but comparable with another local study (33.6%) [13]. The participants in this study were recruited through the health screening programs conducted in 11 states covering most regions in Malaysia, which is the strength of this study. Therefore, the prevalence of dyslipidaemia subtypes, including elevated LDL-C reported in this study, may provide a good representation of the Malaysian population. In addition, this study collected venous blood samples, and the measurement of lipid profiles was performed with a gold-standard laboratory analyser. However, this study has several limitations. First, the majority of the participants in this study were Malays, while other ethnicities (Malaysian Chinese and Indians) were underrepresented. Ideally, a proportional major ethnic group distribution for the country is required for a prevalence study in a multiethnic country such as Malaysia. Secondly, participants’ clinical histories were obtained via face-to-face interviews. Some participants did not remember the age of onset of CAD for their family members or the type of LLT use, which may introduce recall bias. To minimise this, the trained interviewers asked specific questions for clarification, such as the reason for their LLT use and if they have any label or written documentation of their LLT. Third, as this present study recruited participants from the health screening programmes, those with health issues would probably be more interested in participating in the health screening compared to healthy subjects. In addition, the health screening was free of charge. Therefore, they may take this opportunity to check their current health status. As this is a cross-sectional study, the findings in this study could only reflect the relationship but not the causality.

The findings of this study suggest a high prevalence of dyslipidaemia, particularly HC and elevated LDL-C, in the Malaysian community. Despite the high burden of dyslipidaemia, lipid-lowering medication use was low. In addition, the LDL-C target levels achievement, particularly among HR and VHR participants, were still suboptimal. This may suggest a lack of knowledge and awareness of dyslipidaemia among Malaysians and insufficient detection at the community level. The community needs to be educated on dyslipidaemia and its associated morbidity through health campaigns. A well-informed community with adequate health literacy is more likely to be aware of its current health status. Furthermore, adherence to the LLT may be improved with better awareness of dyslipidaemia, including its associated morbidity. The findings of this study emphasised an urgent need to improve the awareness of the general public and healthcare providers on the importance of early detection and efficient treatment of dyslipidaemia, particularly among HR and VHR individuals.

## 5. Conclusions

In conclusion, HC and elevated LDL-C are highly prevalent in the Malaysian community. The majority were undertreated and did not achieve the LDL-C treatment target. Proactive call-to-action programs by stakeholders are warranted to strategize policies and action plans to combat dyslipidaemia-associated CVD events.

## Figures and Tables

**Table 1:** Demographic characteristics of the study population.

Demographic Characteristics	All Subjects (N = 5279)	Male (N = 1996)	Female (N = 3283)
Age (years), mean ± SD	41.1 ± 14.8	43.8 ± 14.8	39.4 ± 14.6
Age group (years), *n* (%)			
18–29	1409 (26.7)	410 (20.5)	999 (30.4)
30–39	1096 (20.8)	391 (19.6)	705 (21.5)
40–49	1091 (20.7)	419 (21.0)	672(20.5)
50–59	1020 (19.3)	465 (23.3)	555 (16.9)
≥60	663 (12.6)	311 (15.6)	352 (10.7)
Ethnicity, *n* (%)			
Malay	3834 (72.6)	1426 (71.4)	2408 (73.3)
Chinese	170 (3.2)	79 (4.0)	91 (2.8)
Indian	101 (1.9)	46 (2.3)	55 (1.7)
Indigenous/others	1174 (22.2)	445 (22.3)	729 (22.2)
Locality, *n* (%)			
Urban	3086 (58.5)	1146 (57.4)	1940 (59.1)
Rural	2193 (41.5)	850 (42.6)	1343 (40.9)
Education attainment*, *n* (%)			
No formal education	229 (4.3)	66 (3.3)	163 (5.0)
Primary education	453 (8.6)	187 (9.4)	266 (8.1)
Secondary education	1684 (32.0)	718 (36.0)	966 (29.4)
Tertiary education	2346 (44.4)	815 (40.8)	1531 (46.6)
Smoking status *, *n* (%)			
Non-smoker	3937 (74.6)	969 (48.5)	2968 (90.4)
Current smoker	655 (12.4)	625 (31.3)	30 (0.9)
Previous smoker	534 (10.1)	344 (17.2)	190 (5.8)
Alcohol consumption, *n* (%)	183 (3.5)	141 (7.0)	42 (1.3)
BMI (kg/m^2^) *, mean ± SD	26.1 ± 5.4	26.2 ± 5.1	26.1 ± 5.6
BMI categories *, [kg/m²], *n* (%)			
Underweight (<18.5)	285 (5.4)	80 (4.0)	205 (6.2)
Normal (18.5–22.9)	1187 (22.5)	411 (20.6)	776 (23.6)
Overweight (23–27.4)	1788 (33.9)	729(36.5)	1059 (32.3)
Obese (≥27.5)	1823 (34.5)	691 (34.6)	1132 (34.5)
Waist circumference (cm), mean ± SD	86.1 ± 13.3	90.4 ± 12.9	83.5 ± 12.8
Hip circumference (cm), mean ± SD	100.3 ± 11.4	99.5 ± 10.3	100.8 ± 12.0
Central obesity *, *n* (%)			
Normal	2186 (44.0)	903 (48.3)	1283 (41.5)
Abdominal obesity (Male ≥90 cm; female ≥80 cm)	2952 (56.0)	1032 (51.7)	1920 (58.5)
Comorbidities *, *n* (%)			
Diabetes	503 (9.5)	250 (12.5)	253 (7.7)
FPG (mmol/L), mean ± SD	5.5 ± 2.5	5.8 ± 3.1	5.3 ± 2.2
RBG (mmol/L), mean ± SD	6.2 ± 3.3	6.6 ± 3.9	5.9 ± 2.9
Hypertension	1662 (31.5)	764 (38.3)	898 (27.3)
SBP (mm/Hg), mean ± SD	124.0 ± 19.3	130.5 ± 17.3	120.0 ± 19.5
DBP (mm/Hg), mean ± SD	76.9 ± 11.7	79.4 ± 11.5	75.4 ± 11.6
Existing CVD	143 (2.7)	82 (4.1)	61 (1.9)
Family history of PCAD, *n* (%)	609 (11.5)	223 (11.2)	386 (11.7)
Lipid profiles, mean ± SD			
TC (mmol/L)	5.2 ± 1.2	5.3 ± 1.3	5.2 ± 1.2
TG (mmol/L)	1.4 ± 0.9	1.7 ± 1.0	1.2 ± 0.8
LDL-C (mmol/L)	3.2 ± 1.0	3.3 ± 1.1	3.1 ± 1.0
HDL-C (mmol/L)	1.3 ± 0.4	1.1 ± 0.4	1.4 ± 0.4
Risk categories, *n* (%)			
Low	3715 (70.4)	1017 (50.9)	2698 (88.2)
Moderate	462 (8.8)	339 (17.0)	123 (3.7)
High	503 (9.5)	345 (17.3)	158 (4.8)
Very high	599 (11.3)	295 (14.8)	304 (9.3)

BMI: body mass index; DBP: diastolic blood pressure; FPG: fasting plasma glucose; RBG: random blood glucose; HDL-C: high-density lipoprotein cholesterol; LDL-C: low-density lipoprotein cholesterol; TC: total cholesterol; TG: triglycerides; PCAD: premature coronary artery disease; SBP: systolic blood pressure; CVD: cardiovascular disease; SD: standard deviation; * Numbers are not equal to 5279 due to missing values.

**Table 2:** Prevalence of dyslipidaemia subtypes according to the demographic and clinical characteristics.

	HC (TC > 5.2 mmol/L)	*p*-Value	Low HDL-C (<1.0 mmol/l [Males] & <1.2 mmol/L [Females])	*p*-Value	High TG (TG >1.7 mmol/L)	*p*-Value	Elevated LDL-C				*p*-Value
							VHR (LDL-C ≥ 1.8 mmol/L)	HR (LDL-C > 2.6 mmol/L)	MR (LDL-C ≥ 3.0 mmol/L)	LR (LDL-C ≥ 3.0 mmol/L)	
All (N = 5279)	2717 (51.5)		1521 (28.8)		1782 (33.8)		517 (9.8)	452 (8.6)	305 (5.8)	1843 (34.9)	
Gender, *n* (%)											
Male	1119 (41.2)	<0.001 *	606 (39.8)	<0.06	967 (54.3)	<0.001 *	260 (50.3)	296 (65.5)	213 (69.8)	523 (28.4)	<0.001 *
Female	1958 (58.8)		915 (60.2)		815 (45.7)		257 (49.7)	156 (34.5)	92 (30.2)	1320 (71.6)	
Age category, *n* (%)											
18–29	395 (14.5)	<0.001 *	288 (18.9)	<0.001 *	193 (10.8)	<0.001 *	20 (3.9)	26 (5.8)	0 (0.0)	497 (27.0)	<0.001 *
30–39	542 (19.9)		354 (23.3)		367 (20.6)		39 (7.5)	44 (9.7)	3 (1.0)	545 (29.6)	
40–49	631 (23.2)		372 (24.5)		439 (24.6)		122 (23.6)	66 (14.6)	80 (26.2)	447 (24.3)	
50–59	702 (25.8)		324 (21.3)		470 (26.4)		184 (35.6)	150 (33.2)	126 (41.3)	288 (15.6)	
≥60	447 (16.5)		183 (12.0)		313 (17.6)		152 (29.4)	166 (36.7)	96 (31.5)	66 (3.6)	
Ethnicity, *n* (%)											
Malay	2069 (76.2)	<0.001 *	1027 (67.5)	<0.001 *	1251 (70.2)	<0.001 *	375 (72.5)	333 (73.7)	215 (70.5)	1429 (77.5)	<0.001 *
Chinese	87 (3.2)		30 (2.0)		67 (3.8)		14 (2.7)	7 (1.5)	8 (2.6)	60 (3.3)	
Indian	67 (2.5)		46 (3.0)		44 (2.5)		21 (4.1)	12 (2.7)	5 (1.6)	35 (1.9)	
Indigenous	494 (18.2)		418 (27.5)		420 (23.6)		107 (20.7)	100 (22.1)	77 (25.2)	319 (17.3)	
Locality, *n* (%)											
Urban	1499 (55.2)	<0.001 *	871 (57.3)	0.120 *	1008 (56.6)	0.013 *	278 (53.8)	231 (51.1)	161 (52.8)	1066 (57.8)	0.028 *
Rural	1218 (44.8)		650 (42.7)		774 (43.4)		239 (46.2)	221 (49.9)	144 (47.2)	777 (42.2)	
Education attainment, *n* (%)											
No formal education	124 (5.2)	0.144 *	75 (5.4)	0.028 *	83 (5.2)	0.011 *	39 (8.5)	27 (7.0)	23 (8.5)	56 (3.4)	<0.001 *
Primary education	73 (3.0)		42 (3.0)		49 (3.1)		17 (3.7)	13 (3.4)	13 (4.8)	40 (2.4)	
Secondary education	178 (7.4)		116 (8.4)		134 (8.4)		55 (12.0)	45 (11.7)	33 (12.2)	69 (4.2)	
Tertiary education	2032 (84.4)		1155 (83.2)		1327 (83.3)		346 (75.7)	298 (77.8)	202 (74.5)	1490 (90)	
Smoking status, *n* (%)											
Non-smoker	2012 (74.1)	<0.001 *	1160 (76.3)	<0.001 *	1214 (68.1)	<0.001 *	344 (66.5)	232 (51.3)	186 (61.0)	1553 (84.3)	<0.001 *
Current smoker	374 (13.8)		245 (16.1)		350 (19.6)		95 (18.4)	142 (31.4)	75 (24.6)	130 (7.1)	
Previous smoker	331 (12.2)		116 (7.6)		218 (12.2)		78 (15.1)	78 (17.3)	44 (14.4)	160 (8.7)	
BMI categories, *n* (%)											
Underweight (<18.5)	81 (3.1)	<0.001 *	25 (1.7)	<0.001 *	21 (1.2)	<0.001 *	6 (1.2)	12 (2.7)	5 (1.7)	75 (4.2)	<0.001 *
Normal (18.5–22.9)	464 (17.8)		196 (13.2)		200 (11.6)		38 (7.6)	53 (12.0)	62 (21.6)	386 (21.8)	
Overweight (23–27.4)	1001 (38.5)		507 (34.2)		653 (38.0)		186 (37.3)	203 (46.1)	115 (40.1)	646 (36.5)	
Obese (≥27.5)	1057 (40.6)		755 (50.9)		843 (49.1)		269 (53.9)	172 (39.1)	105 (36.6)	663 (37.5)	
Central obesity, *n* (%)											
Normal	909 (34.3)	<0.001 *	393 (26.5)	<0.001	457 (26.4)	<0.001 *	97 (19.4)	136 (30.8)	107 (36.5)	718 (40.0)	<0.001 *
Abdominal obesity	1738 (65.7)		1090 (73.5)		1272 (73.6)		404 (80.6)	306 (69.2)	186 (63.5)	1079 (60.0)	

HC = Hypercholesterolaemia; TC = Total cholesterol; LDL-C = Low-density lipoprotein cholesterol; TG = Triglyceride; HDL-C = High-density lipoprotein cholesterol; VHR = Very high risk; HR = High risk; MR = Moderate risk; LR = Low risk. * Pearson chi-square.

**Table 3:** Proportions of LLT use and the achievements of LDL-C targets according to risk categories.

Risk Categories	On LLT, *n* (%)		Total	χ² Value (df)	*p*-Value ^	LDL-C Target Levels Achievement +, *n* (%)		Total	χ² Value (df)	*p*-Value ^
	Yes	No				Achieved	Not Achieved			
Low (LR)	94 (2.5)	3621 (97.5)	3715 (70.4)	446.1 (3)	<0.001	42 (44.7)	52 (55.3)	94 (26.1)	30.2 (3)	<0.001
Moderate (MR)	59 (12.8)	403 (87.2)	462 (8.8)			21 (35.6)	38 (64.4)	59 (16.4)		
High (HR)	62 (12.3)	441 (87.7)	503 (9.5)			10 (16.1)	52 (83.9)	62 (17.2)		
Very high (VHR)	145 (24.2)	454 (75.8)	599 (11.3)			23 (15.9)	122 (84.1)	145 (40.3)		
i. CVD	44 (30.8)	99 (69.2)	143 (23.9)			5 (11.4)	39 (88.6)	44 (30.3)		
ii. DM with risk factors	101 (22.1)	355 (77.9)	456 (76.1)			18 (17.8)	83 (82.2)	101 (69.7)		
Total	360 (6.8)	4919 (93.2)	5279 (100)	-	-	96 (26.7)	264 (73.3)	360 (100)	-	-

^ Pearson chi-square. + For participants on LLT: LDL-C target for low risk < 3.0 mmol/L, moderate risk < 3.0 mmol/L, high risk ≤ 2.6 mmol/L and very high risk < 1.8 mmol/L; df: degree of freedom; LLT: lipid-lowering drug; LDL-C: low density lipoprotein-cholesterol; CVD: cardiovascular disease; DM: diabetes mellitus.

## Data Availability

Not applicable.

## Appendix A. Malaysian HEalth and Well-Being AssessmenT for Coronary Risk Epidemiological Study (MyHEBAT-CRES) Research Investigators

**UNIVERSITI TEKNOLOGI MARA (UiTM):** Hapizah Nawawi (I-PPerForM, Faculty of Medicine and Al-Sultan Abdullah Hospital), Anis Safura Ramli (I-PPerForM and Faculty of Medicine), Suraya Abdul Razak (I-PPerForM and Faculty of Medicine), Noor Alicezah Mohd Kasim (I-PPerForM, Faculty of Medicine and Al-Sultan Abdullah Hospital), Thuhairah Hasrah Abdul Rahman (I-PPerForM, Faculty of Medicine and Al-Sultan Abdullah Hospital), Effat Omar (I-PPerForM, Faculty of Medicine and Al-Sultan Abdullah Hospital), Noor Shafina Mohd Nor (I-PPerForM, Faculty of Medicine and Al-Sultan Abdullah Hospital), Suhaila Abd Muid (I-PPerForM and Faculty of Medicine), Mansharan Kaur Chainchel Singh (I-PPerForM, Faculty of Medicine and Al-Sultan Abdullah Hospital), Yung-An Chua (I-PPerForM), Nurul’Ain Abu Bakar (I-PPerForM), Aimi Zafira Razman (I-PPerForM), Sukma Azureen Nazli (I-PPerForM), Amirah Mohd Ariff (I-PPerForM), Fadlullah Jili Fursani Kemrry (I-PPerForM), Mohd Yusmiaidil Putera Mohd Yusof (I-PPerForM and Faculty of Dentistry), Zaliha Ismail (Faculty of Medicine), Norashikin Mohd Ranai (Faculty of Medicine and Al-Sultan Abdullah Hospital), Ahmad Taufik Jamil (Faculty of Medicine and Al-Sultan Abdullah Hospital), Ahmad Bakhtiar Md Radzi (Faculty of Medicine and Al-Sultan Abdullah Hospital), Khairul Shafiq Ibrahim (Faculty of Medicine and Al-Sultan Abdullah Hospital), Zalizah Khalid (Faculty of Medicine and Al-Sultan Abdullah Hospital), Fatmawati Kamal (Faculty of Medicine and Al-Sultan Abdullah Hospital), Wan Asmuni Wan Mohd (Faculty of Medicine and Al-Sultan Abdullah Hospital), Nurul Alimah Abdul Nasir (Faculty of Medicine), Arjoanna Farra Azizi (Faculty of Medicine and Al-Sultan Abdullah Hospital), Mohamad Rodi Isa (Faculty of Medicine), Mohd Shahril Ahmad Saman (Faculty of Medicine), Akmal Zulayla Mohd Zahid (Faculty of Medicine and Al-Sultan Abdullah Hospital), Mohammad Hanafiah Kreah (Faculty of Medicine and Al-Sultan Abdullah Hospital), Mohd Izwan Mat Nazali (Faculty of Medicine), Madyhah Abd. Monir (Faculty of Medicine and Al-Sultan Abdullah Hospital), Fathimah Mohamad (Faculty of Medicine and Al-Sultan Abdullah Hospital), Nadzimah Mohd Nasir (Faculty of Medicine and Al-Sultan Abdullah Hospital), Noor Azean Anis Abd Aziz (Faculty of Medicine and Al-Sultan Abdullah Hospital), Norashikin Mohd Tambeh (Faculty of Medicine and Al-Sultan Abdullah Hospital), Fadzilah Mohd Noor (Faculty of Medicine), Syahrul Azlin Shaari (Faculty of Medicine), Siti Munira Yasin (Faculty of Medicine). **UNIVERSITY OF MALAYA (UM):** Mohd Amin Jalaludin (Faculty of Medicine and University Malaya Medical Centre); **UNIVERSITI SULTAN ZAINAL ABIDIN (UniSZA):** Ahmad Zubaidi Abdul Latif (Faculty of Medicine and UnisZA Teaching Hospital); **UNIVERSITI KUALA LUMPUR (UniKL):** Osman Ali (Royal College of Medicine Perak).
